# Hyperforin and Myrtucommulone Derivatives Act as Natural Modulators of Wnt/β-Catenin Signaling in HCT116 Colon Cancer Cells

**DOI:** 10.3390/ijms23062984

**Published:** 2022-03-10

**Authors:** Aneliya Knauthe, Sonnhild Mittag, Laura Bloch, Kai Frederik Albring, Martin Schmidt, Oliver Werz, Otmar Huber

**Affiliations:** 1 Institute of Biochemistry II, Jena University Hospital, Friedrich Schiller University Jena, 07743 Jena, Germany; aneliya.knauthe@gmail.com (A.K.); sonnhild.mittag@med.uni-jena.de (S.M.); laura_bloch@outlook.de (L.B.); kai.albring@yahoo.de (K.F.A.); martin.schmidt@med.uni-jena.de (M.S.); 2 Department of Pharmaceutical/Medicinal Chemistry, Institute of Pharmacy, Friedrich Schiller University Jena, 07743 Jena, Germany; oliver.werz@uni-jena.de

**Keywords:** hyperforin, myrtucommulone, Wnt/β-catenin signaling, cell proliferation, colony formation, *Myrtus communis*, *Hypericum perforatum* L. (St. John’s wort), acylphloroglucinol

## Abstract

The therapeutic activities of natural plant extracts have been well known for centuries. Many of them, in addition to antiviral and antibiotic effects, turned out to have anti-tumor activities by targeting different signaling pathways. The canonical Wnt pathway represents a major tumorigenic pathway deregulated in numerous tumor entities, including colon cancer. Here, we investigated the acylphloroglucinols hyperforin (HF) from St. John’s wort (*Hypericum perforatum* L.) and myrtucommulone A (MC A) from myrtle (*Myrtus communis*) and semi-synthetic derivatives thereof (HM 177, HM 297, HM298) for their effects on Wnt/β-catenin signaling. None of these substances revealed major cytotoxicity on STF293 embryonic kidney and HCT116 colon carcinoma cells at concentrations up to 10 μM. At this concentration, HF and HM 177 showed the strongest effect on cell proliferation, whereas MC A and HM 177 most prominently inhibited anchorage-independent growth of HCT116 cells. Western blot analyses of active β-catenin and β-catenin/TCF reporter gene assays in STF293 cells revealed inhibitory activities of HF, MC A and HM 177. In line with this, the expression of endogenous Wnt target genes, Axin and Sp5, in HCT116 cells was significantly reduced. Our data suggest that the acylphloroglucinols hyperforin, myrtucommulone A and its derivative HM 177 represent potential new therapeutic agents to inhibit Wnt/β-catenin signaling in colon cancer.

## 1. Introduction

Extracts from medical plants and herbs have a long history in traditional medicine, and a significant part of the world population relies on the therapeutic and preventive effects of plant-derived substances for health care. Up to now, many of the physiologically active compounds within plant extracts have been unknown, but interest in these compounds is constantly rising. With improvement in separation tools and analytical methods, the number of known active substances and their chemical structure is growing. Subsequent successful chemical synthesis allows for obtaining sufficient amounts of the substances of interest for detailed studies on their biological activities. Among these plant-derived substances, hyperforin (HF) from St. John’s wort (SJW) (*Hypericum perforatum* L.) and myrtucommulone A (MC A) from myrtle (*Myrtus communis*) have attracted a lot of attention in respect to their broad spectrum of pharmacological effects. However, the molecular mechanisms behind these effects are currently not completely understood.

**Figure 1:**
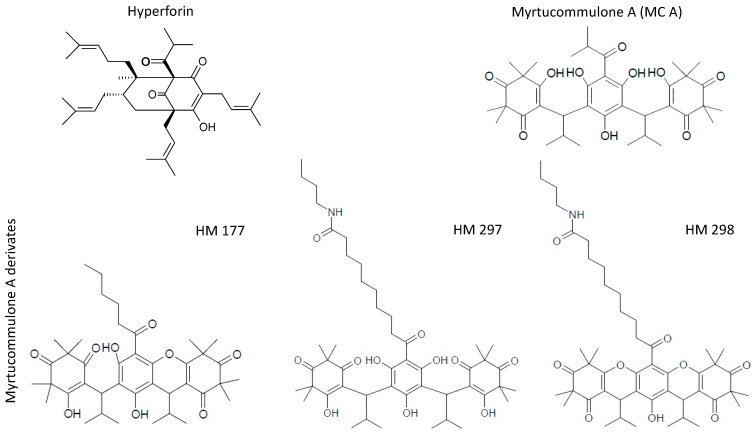
Chemical structures of compounds used in this study.

Both HF (CAS number: 11079-53-1) and MC A (CAS number: 54247-21-1) represent acylphloroglucinol derivatives (for chemical structures, see Figure 1) with reported anti-oxidative, anti-inflammatory, anti-viral, anti-bacterial, anti-fungal, anti-diabetic, hepato- and neuroprotective and anti-cancer properties [1,2]. Of the many bioactive compounds detected in St. John’s wort and other *Hypericum* species [3], HF is by far the most abundant and characteristic ingredient for this species, in addition to biapigenin, adhyperforin, pseudohypericin, hypericin and quercetins [4]. HF together with hypericin have attracted a lot of attention in respect to their potential anti-depressant effects and their therapeutic use [5]. Corresponding investigations include studies on the serum levels that can be obtained by oral administration [6]. Similar to *Hypericum* species, members of the family of *Myrtacea* are well known for their biologically active constituents, including terpenoids, flavonoids, such as quercetin, catechin and myricetin derivatives [1], as well as myrtucommulones (MCs) and related acylphloroglucinols [7], which reveal a wide spectrum of pharmacological effects. The antibacterial activity of MC A had already been reported nearly 50 years ago [8].

In recent years, the anti-inflammatory and anti-cancer activities of HF and myrtucommulones have also come into focus (for review, see [7,9,10]). In this respect, HF has been shown to act as a potent cyclooxygenase-1 and 5-lipoxygenase inhibitor [11,12]. In addition, it inhibits prostaglandin E_2_ synthase-1 [13,14]. As an additional mechanism of action, the inhibition of inflammation-induced activation of STAT-1/-3 transcription factors NF-κB and MAPKs by HF was reported (for review, see [15] and references therein). Similarly, MC A shows inhibitory activity on cyclooxygenase-1, 5-lipoxygenase and prostaglandin E_2_ synthase-1 in vitro and in vivo [16,17,18,19]. The anti-tumor activities of HF and myrtucommulones appear to depend on different signaling pathways and targets involved in processes such as cell proliferation and apoptosis [19,20,21,22,23,24,25], cell migration, invasion and metastasis [26,27], and genotoxicity [28].

Oral bioavailability of HF and MC A suggests that the intestinal epithelium is the primary site of uptake. Thus, the anti-cancer and anti-inflammatory actions of these compounds should also be active in the gastrointestinal tract. Interestingly, Satia et al. reported that continuous use of SJW extract reduces the risk of colon cancer by 65% [29]. In line with this observation, in an azoxymethane-induced colorectal carcinogenesis model, dietary supplementation with SJW extract resulted in decreased number and size of tumors. This effect was attributed to an attenuated proinflammatory signaling in response to inhibition of NF-κB and ERK1/2 pathways [30]. In a case report, beneficial effects of SJW extract were reported in two cases of colon cancer and one duodenal adenocarcinoma [31]. These observations suggest that HF might have a beneficial therapeutic effect in colorectal cancer treatment. To our knowledge, nothing is known about myrtucommulones in this respect.

It is well known that the canonical Wnt signaling pathway plays a central role during colorectal carcinogenesis. In up to 80% of spontaneous colorectal cancer cases, mutations of the adenomatous polyposis coli (APC) tumor suppressor gene induce an activation of the Wnt pathway. In consequence, β-catenin accumulates in the cytosol and nucleus and activates transcription of target genes, driving tumorigenesis. In healthy cells, excess β-catenin is rapidly phosphorylated within the β-catenin destruction complex composed of APC, Axin, casein kinase 1 (CK1) and glycogen synthase kinase-3β (GSK-3β). Both kinases phosphorylate β-catenin at Ser/Thr residues within its N-terminus, marking it for ubiquitination by the β-TRCP (β-transducin repeat containing E3 ubiquitin protein ligase) complex and subsequent proteasomal degradation (for review, see [32]). This makes the Wnt signaling pathway an attractive target for treatment of colorectal cancer [33]. In this context, plant-derived substances, such as curcumin [34,35] or berberine [36,37], have been reported to inhibit Wnt/β-catenin signaling (for review, see [38]).

Here, we address the question of whether HF and MC A and derivatives HM 177, HM 297 and HM 298 thereof are able to attenuate Wnt/β-catenin signaling in Wnt3a-stimulated HEK-293 cells and HCT116 colon cancer cells. MC A derivatives HM 177, HM 297 and HM 298 were chosen from our previous structure–activity relationship evaluation that identified HM 177 and HM 297 as potent MC A derivatives and as suitable tools for mechanistic studies on intrinsic mitochondrial pathways, where HM 298 served as negative control for HM 297 [19,21,39]. We show that HF and MCs exhibit an anti-proliferative effect on HCT116 cells and inhibit anchorage-independent growth. Reporter gene assays revealed reduced β-catenin transcriptional activity, and, consistently, the expression of endogenous β-catenin target genes is down-regulated. Taken together, these observations indicate that the anti-tumor activity of HF and MCs is at least in part mediated by an attenuation of the canonical Wnt signaling pathway.

## 2. Results

### 2.1. Cytotoxicity of HF and Myrtucommulones and Their Effects on Cell Viability

To define the concentration range that can be applied for subsequent functional studies of HF, MC A and its derivatives HM 177, HM 297 and HM 298 (see Figure 1 for chemical structures), cytotoxicity and viability assays were performed with STF293 cells (HEK-293 cells stably transfected with the SuperTop-Flash Wnt-reporter) and with HCT116 colon carcinoma cells characterized by an intrinsically active Wnt signaling pathway due to a mutation in the β-catenin N-terminal phosphorylation motif.

**Figure 2:**
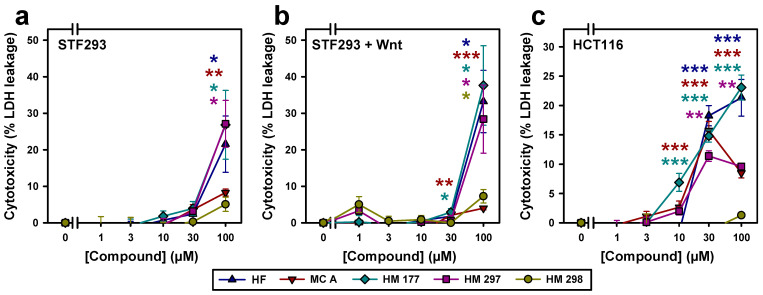
Cytotoxicity of HF, MC A and its derivatives HM 177, HM 297 and HM 298, as analyzed by quantification of lactate-dehydrogenase released in response to the indicated concentrations of compounds from (**a**) STF293 (*n* ≥ 3; two-tailed Student’s *t*-test), (**b**) STF293 cells treated with Wnt3a-conditioned medium (*n* = 3 for HF, MC A, HM177; *n* = 2 for HM 297 and HM 298; two-tailed Student’s *t*-test) and (**c**) HCT116 cells (*n* ≥ 6; Mann–Whitney rank sum test) normalized to DMSO solvent control. Each sample was measured in duplicate. * *p* < 0.05 ** *p* < 0.01; *** *p* < 0.001.

Cytotoxicity was measured at concentrations of 1, 3, 10, 30 and 100 μM of the compounds based on the release of lactate-dehydrogenase as previously described [36]. In STF293 cells, minor cytotoxicity was detectable in response to HF, MC A, HM 177 and HM 297 treatment up to concentrations of 30 μM. HF, HM 177 and HM 297 turned to reveal cytotoxic effects at 100 μM of the compounds. The proposed inactive ring-closed analog of HM 297, namely HM 298, showed only minor cytotoxicity even at the highest tested concentration (Figure 2a), as expected [21]. After treatment of STF293 cells with Wnt3a-conditioned medium, the substances revealed similar cytotoxic effects (Figure 2b). Interestingly, compared to STF293 cells, in HCT116 cells, evidently higher cytotoxic effects were detected for HF, MC A and HM 177 at a concentration of 30 μM. Even HM 297 showed a cytotoxic effect, starting at 30 μM. Despite an obvious higher sensitivity of HCT116 cells, no cytotoxicity was detectable for HM 298 (Figure 2c).

**Figure 3:**
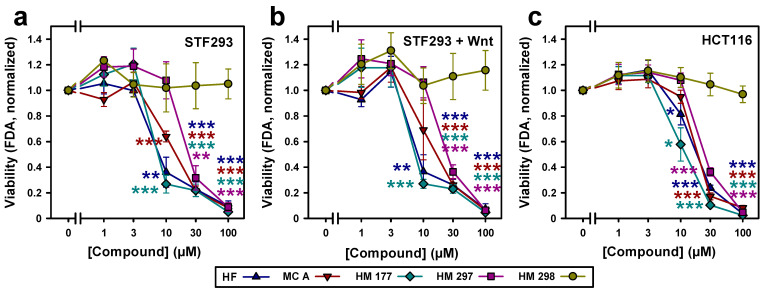
Analysis of cell viability in response to treatment with the indicated concentrations of HF, MC A and its derivatives HM 177, HM 297 and HM 298 on (**a**) STF293 (*n* = 3; two-tailed Student’s *t*-test) and (**b**) STF293 cells after stimulation with Wnt3a-conditioned medium (*n* = 3; two-tailed Student’s *t*-test) and (**c**) HCT116 cells (*n* = 7; Mann–Whitney rank sum test) using FDA assays. Each value was measured in duplicate. * *p* < 0.05 ** *p* < 0.01; *** *p* < 0.001.

In complementary fluorescein diacetate (FDA) viability assays quantifying metabolically active cells, concentrations up to 3 μM of HF, MC A, HM 177 and HM 297 did not show major reduction in cell viability in STF293 cells. HF, MC A and HM 177 affected viability at 10 μM, whereas HM 297 affected viability at 30 μM. HM 298 did not exhibit any effects up to 100 µM (Figure 3a). Similar results were obtained for STF293 after Wnt3a stimulation (Figure 3b). In HCT116 cells, HF and HM 177 significantly impaired viability at a concentration of 10 μM. This effect appears to be not as strong as in STF293 cells. MC A and HM 297 attenuated viability at 30 μM and higher concentration. As for STF293 cells, HM 298 showed no effect on HCT116 viability (Figure 3c).

### 2.2. HF and HM 177 Exhibit Strong Anti-Proliferative Effect

**Figure 4:**
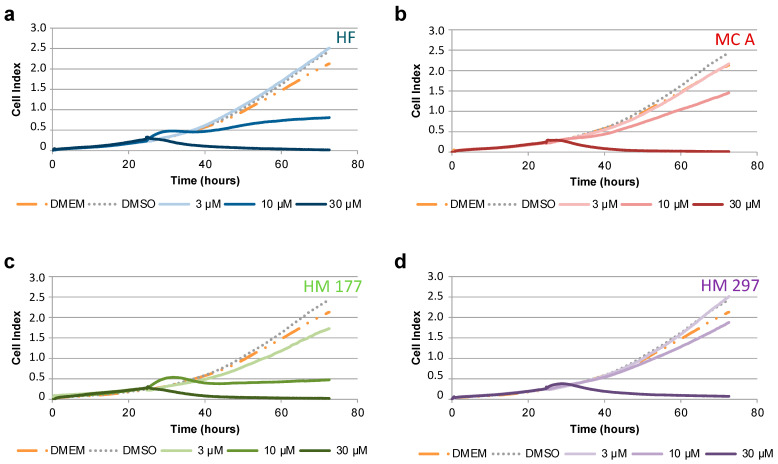
Analysis of cell proliferation of HCT116 cells using real-time impedimetric measurement using the xCELLigence system in response to treatment with the indicated concentrations of (**a**) HF, (**b**) MC A and its derivatives (**c**) HM 177 and (**d**) HM 297. The graphs show representative cell index values of experiments performed in duplicate.

The anti-proliferative activity of HF and MCs on HCT116 colon cancer cells was quantified using the real-time xCELLigence cell analyzer. After setting and adaption of cells for 24 h, compounds were added at the indicated concentrations, and cells were analyzed for further 48 h. Cell index values were compared with untreated cells in DMEM or with cells treated with 0.1% (*v*/*v*) DMSO vehicle control. At the highest tested concentration of 30 μM, all analyzed compounds (HF, MC A, HM 177 and HM 297) stopped cell proliferation. It cannot be excluded that cytotoxic effects contributed to this fact. At a concentration of 10 μM, the strongest reduction in cell proliferation was detected after addition of HF and HM 177. MC A revealed a clearly weaker anti-proliferative effect, and HM 297 also exhibited only minor activity (Figure 4). HM 298 was not tested because it did not show effects in previous assays. Similar results were obtained for STF293 cells (Appendix A).

### 2.3. MC A and HM 177 Strongly Inhibit Anchorage-Independent Growth

**Figure 5:**
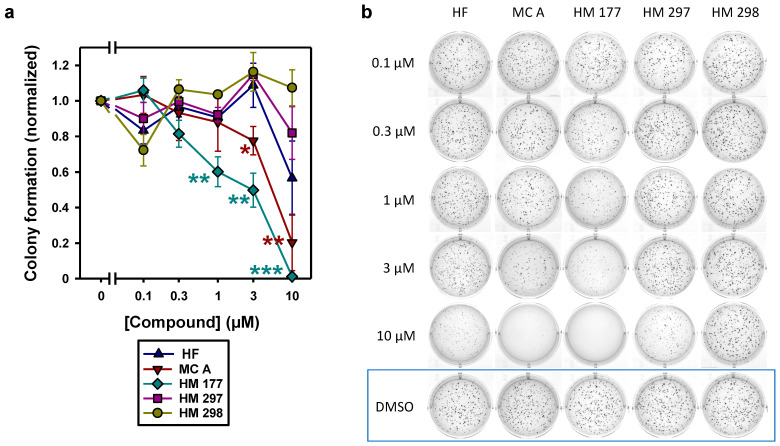
Anchorage-independent growth of HCT116 cells was analyzed by soft agar colony formation assays. HF and MCs were added at the indicated concentrations, and cells were grown for 21 days. Relative number of colonies normalized to DMSO control are shown. (**a**) Results of 3 independent experiments, each performed in triplicate, are summarized in the graph. * *p* < 0.05; ** *p* < 0.01; *** *p* < 0.001 (two-tailed Student’s *t*-test). (**b**) Images of colonies as taken from a representative experiment.

A typical characteristic of tumor cells is their ability to divide independent of contact to extracellular matrix or neighboring cells. To test whether HF and myrtucommulone compounds are able to impair anchorage-independent growth, HCT116 cells were seeded as single cells in soft agar and analyzed for colony formation. In general, it was noted that with increasing concentrations of the compounds, not only the number of colonies was markedly affected, but also their size. Interestingly, in this type of assay, HM 177 and MC A showed the strongest inhibitory activity. A significant concentration-dependent effect was detectable starting at only 1 μM for HM 177 and at 3 μM for MC A. An increase to 10 μM resulted in a drastic reduction in soft agar growth, probably due to triggering cytotoxic side effects and a strong increase in the number of colonies falling below the threshold set for detection as a colony. An inhibitory activity for HF was first detectable at a concentration of 10 μM, but it did not reach significance. HM 297 revealed an only minor effect at the highest tested concentration of 10 μM (Figure 5a). Surprisingly, increasing concentrations of HM 298 tended to form even more, but rather smaller, colonies (Figure 5b).

### 2.4. HF and MCs Repress Canonical Wnt Signaling

**Figure 6:**
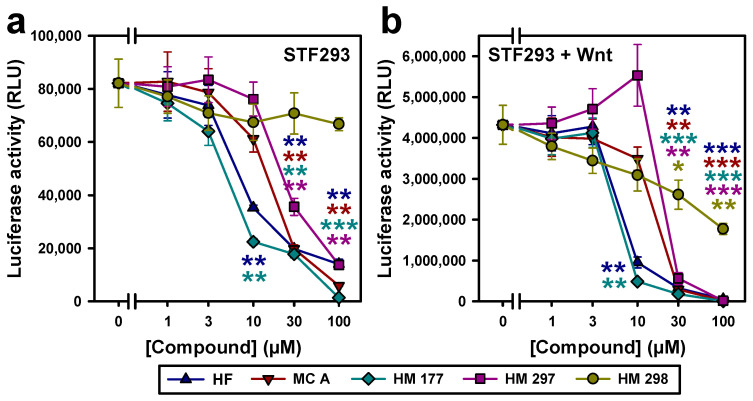
HF and MCs inhibit canonical Wnt signaling, as analyzed in reporter gene assays with (**a**) STF293 cells (**b**) or Wnt3a-conditioned-medium-treated STF293 cells. Relative luminescence units (RLU) were measured under identical conditions. The graph summarizes results of 3 independent experiments measured in duplicate. * *p* < 0.05; ** *p* < 0.01, *** *p* < 0.001 (two-tailed Student’s *t*-test).

Cell proliferation and anchorage-independent growth is known to be promoted by the Wnt/β-catenin signaling [40]. Thus, in the next set of experiments, we wanted to figure out whether the anti-proliferative and colony growth inhibitory activity of HF and MCs is at least in part mediated by an inhibitory effect on the canonical Wnt pathway. To test this, reporter gene assays were performed in STF293 cells, carrying a stably transfected Topflash Wnt reporter construct with seven TCF/LEF consensus binding sites driving firefly luciferase expression [41]. HF and HM 177 induced a significant reduction in reporter gene activity at 10 µM, with HM 177 showing a stronger effect. At 30 μM, MC A and HM 297 also showed significant inhibition of luciferase activity. HM 298 only induced a minor effect, not reaching the limit of significance even at a concentration of 100 μM (Figure 6a). These values were compared to STF293 cells stimulated with Wnt3a-conditioned medium before treatment with different concentrations of compounds. Wnt-conditioned medium, as expected, induced a strong increase in luciferase activity, as shown by the luminescence units depicted on the x-axis. Again, HF and HM 177 most effectively lowered the reporter gene activity. HM 297 exhibited a somehow unexpected behavior in increasing luminescence units at concentrations up to 10 μM and a subsequent strong reduction at 30 μM. Interestingly, HM 298 also revealed an inhibitory activity in the presence of Wnt3a (Figure 6b).

**Figure 7:**
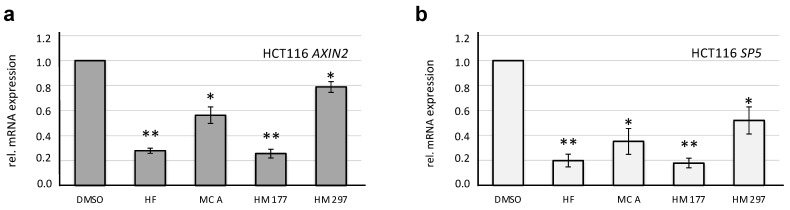
HF and MCs down-regulate mRNA expression of the Wnt target genes *Axin2* (**a**) and *Sp5* (**b**) in HCT116 cells. Compounds were added at a concentration of 3 μM for 20 h before total RNA was isolated and used for qRT-PCR. GAPDH was used as a housekeeping gene for normalization to the vehicle (0.1% (*v*/*v*) DMSO) control. The graph presents the results of 3 independent experiments, each measured in duplicate. * *p* < 0.05; ** *p* < 0.01.

An inhibitory activity of HF and the MCs on the canonical Wnt pathway as detected in the reporter gene assays should also result in the down-regulation of endogenous Wnt target gene expression. Due to a stabilizing mutation in β-catenin, the canonical Wnt pathway in these cells is intrinsically activated. To evaluate this in more detail, quantitative RT-PCR was performed on RNA isolated from HCT116 cells to determine the expression of the well-known Wnt target genes *Axin2* [42,43] and *SP5* [44,45], encoding a component of the β-catenin destruction complex and a Zn finger transcription factor, respectively. HCT116 cells were treated with 3 μM of compounds to avoid toxic side effects. HF and HM 177 induced a strong down-regulation of both target genes, whereas MC A and HM 297 showed weaker effects (Figure 7). Taken together, these data further point to a Wnt inhibitory effect of the compounds.

**Figure 8:**
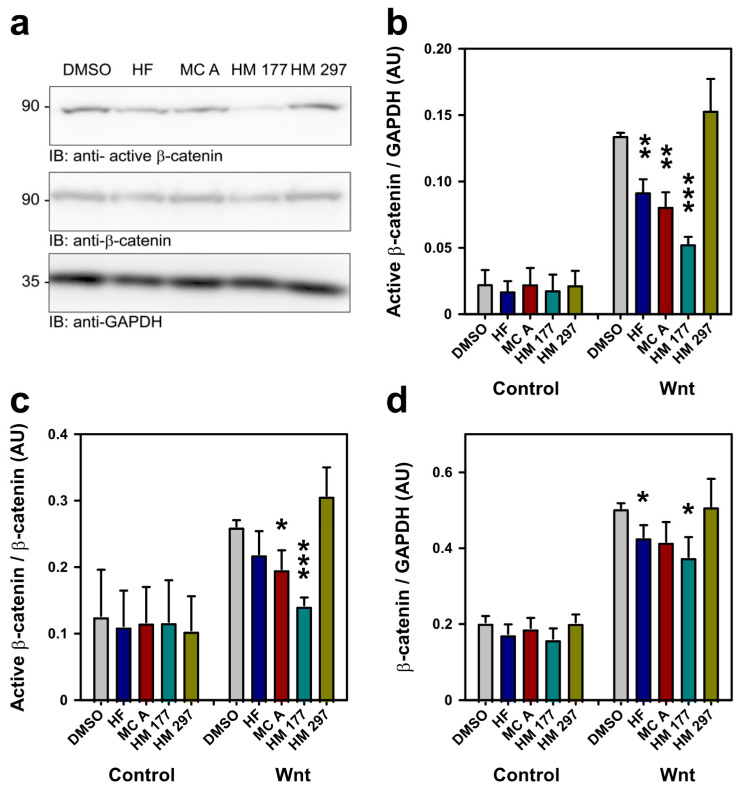
Down-regulation of active β-catenin in response to treatment with HF and MCs. STF293 cells were stimulated with Wnt3a-conditioned medium for 6 h before compounds were added at 3 μM for further 20 h. Cell lysates were analyzed by SDS-PAGE and Western blotting with the indicated antibodies. (**a**) Representative Western blot analysis of active β-catenin, total β-catenin and GAPDH (loading control). Quantification of chemiluminescence signals depicted as (**b**) active β-catenin relative to GAPDH levels, (**c**) active β-catenin to total β-catenin levels or (**d**) total β-catenin to GAPDH levels. Results were obtained from 6 independent experiments. * *p* < 0.05; ** *p* < 0.01; *** *p* < 0.001 (paired Student’s *t*-test).

In the next step, we wanted to test whether the compound-induced reduction in Wnt target gene expression correlated with reduced amounts of active, non-phosphorylated β-catenin. STF293 cells were stimulated with Wnt3a-conditioned medium and subsequently treated with 3 μM HF, MC A, HM 177 and HM 297. Cell lysates were analyzed by SDS-PAGE and Western blotting with anti-active β-catenin antibody and compared with an antibody detecting total β-catenin (clone 14) and anti-GAPDH as loading controls (Figure 8a). As quantified in Figure 8b–d, HF, MC A and HM 177 induced a significant down-regulation of active β-catenin levels relative to GAPDH loading control compared to the vehicle control (Figure 8b). When we compared active β-catenin relative to total β-catenin only, MC A and HM 177 showed significant effects (Figure 8c). In addition, we also tested total β-catenin levels relative to GAPDH loading control, revealing a minor reduction in total β-catenin levels reaching significance for HF and HM 177 treatment (Figure 8d). Similar results were obtained in Western blot analyses of active β-catenin and β-catenin in HCT116 cells, where, in addition, *cyclin D1* and *c-myc* expression was analyzed (Appendix B). Taken together, these data suggest that MC A and HM 177 treatment reduces active β-catenin levels to a higher degree as compared to a concomitant down-regulation of total β-catenin. HM 297 did not show any effect on either active β-catenin or total β-catenin levels.

## 3. Discussion

In ancient medicine, plant-derived extracts played an essential role to treat infections, wounds, metabolic disorders and other diseases. In recent years, more and more pharmacological active compounds of natural origin were identified, specifically from plant extracts. Among these, hyperforin (HF) isolated from *Hypericum perforatum* L. (SJW) and myrtucommulone A (MC A) from *Myrtus communis* have attracted attention. Specifically, HF, which is considered an active ingredient of SJW for the therapy of mild and moderate depressive disorders [46], was put into focus. In addition, the anti-inflammatory and anti-tumor activities of both HF and MC A raised interest in these compounds and derivatives thereof.

In this study, we used STF293 and HCT116 cells to investigate whether the anti-tumor activity of HF, as well as MC A and its derivatives HM 177 and HM 297, can be attributed to an inhibition of canonical Wnt/β-catenin signaling, an oncogenic pathway contributing to tumor formation in many different tissues, e.g., colon cancer. In most cases, the pathway is inappropriately activated by mutations of pathway components (e.g., APC, Axin, β-catenin, RNF43) or of modulators thereof (e.g., Bcl-9, Pygo), resulting in enhanced transcription of Wnt target genes mediated by the β-catenin/TCF transcription complex [33].

At low concentrations of 1 and 3 μM, all tested substances did not affect the viability of both STF293, Wnt3a-treated STF293 and HCT116 cells in FDA assays. In respect to cytotoxicity, concentrations up to 10 μM did not show major effect with HM 177, revealing minor cytotoxic effects in HCT116 cells. When we analyzed growth behavior by real time impedance measurements, using the xCELLigence system, HF and HM 177 revealed a strong inhibitory effect at 10 μM, whereas MC A was less effective, and HM 297 did not show a major difference. At a concentration of 30 μM, a strong growth inhibitory effect was detectable for all four substances. Interestingly, in anchorage-independent growth assays, representing a more physiological situation, HM 177 already induced a significant reduction in colony formation at a concentration of 1 μM. MC A also showed a clear growth inhibitory activity, however weaker than HM 177. Surprisingly, HF, in this type of assay, only showed a minor effect at a concentration of 10 μM, whereas in most other assays, HF belonged to the most active compounds and already showed significant effects at lower concentrations. We currently do not know what causes this difference. We hypothesize that anchorage-independent growth is regulated by signaling pathways that may be affected by HF only in an indirect way.

When testing the substances for their Wnt inhibitory activity, Wnt reporter gene assays in STF293 cells carrying a stably integrated Wnt reporter construct revealed that HF, MC A and HM 177 indeed repress expression of the luciferase reporter gene, both in unstimulated and Wnt3a-stimulated cells. This repressive activity was also detectable in HCT116 cells when we analyzed the expression of the endogenous Wnt target genes *Axin2* and *SP5*. The strongest effects were obvious in response to HF and HM 177 treatment, leading to a 75% reduction in both target genes. Furthermore, the levels of active β-catenin were significantly reduced by HF, MC A and HM177, although a reduction in total β-catenin was also detectable. This, again, indicates that these substances have Wnt inhibitory activities.

Future studies have to unravel whether HF, MC A and its derivative HM 177 directly or indirectly modulate the canonical Wnt signaling pathway in colon cancer cells. Based on previous reports in the literature, Akt1 kinase might play a role. In this respect, Merhi et al. showed that HF directly inhibits Akt1 kinase activity in AML cells with an IC_50_ value of 2.5 μM [47], a concentration where HF also shows Wnt-inhibitory function in our study. The inhibition of Akt activity may reduce inhibitory GSK-3β phosphorylation, and thus lead to enhanced degradation of β-catenin, as observed in our study. Preliminary studies have revealed that GSK-3β kinase activity was not affected by MCs in in vitro kinase assays. In addition, Akt can phosphorylate β-catenin-Ser552, promoting its transcriptional activity [48]. In this context, a decrease in phospho-β-catenin-Ser552 in response to two days of treatment with 25 μM MC A, as observed in bladder and mammary gland cancer cells [49], may contribute to a reduced transcription of β-catenin target genes. In addition, it was observed that MC A also affects phosphorylation of β-catenin on Ser675 in human mesenchymal stem cells [50]. This, in consequence, might also repress β-catenin transcriptional activity. Moreover, it has to be considered that HF and MC A and derivatives thereof might also affect β-catenin signaling activity by their inhibitory activity on microsomal prostaglandin E_2_ synthase-1 activity [13,19]. It has been reported that prostaglandin E_2_, in binding to its receptor EP2, activates stimulatory G proteins. The activated Gα_s_ subunits release GSK-3β from Axin within the β-catenin degradation complex, thereby abrogating β-catenin degradation. In addition, Gβγ subunits stimulate PI3K and Akt, leading to inhibitory phosphorylation of GSK-3β, again, resulting in β-catenin stabilization [51]. Thus, the inhibition of microsomal prostaglandin E_2_ synthase-1 by HF and MC A may contribute to a down-regulation of β-catenin signaling activity.

Taken together, this study shows that HF, MC A and its derivative HM 177 reduce HCT116 colon cancer cell proliferation and anchorage-independent growth. Moreover, our results suggest that these effects are at least in part mediated by an inhibition of the canonical Wnt signaling pathway. Further studies are needed to define the potential crosstalk with other signaling pathways and to determine whether HF and MCs are also able to inhibit the Wnt pathway in vivo. In this respect, the encapsulation of HF into acetalated dextran polymeric nanoparticles may improve its uptake and thus bioactivity, due to reduced plasma protein binding, as shown recently for neutrophils [52].

## 4. Materials and Methods

### 4.1. Compounds

MC A and its derivatives HM 177, HM 297 and HM 298 were designed, synthesized and characterized by Dr. Hans Müller and Prof. Dr. Johann Jauch, Organic Chemistry II, Saarland University, Germany, as described previously [53,54] and received as generous gifts. Hyperforin-dicyclohexammonium (DCHA) salt was obtained from Dr. Willmar Schwabe GmbH & Co. KG (Karlsruhe, Germany) as generous gift. All compounds were dissolved as 30 mM stock solutions in DMSO and were blinded for initial experiments.

### 4.2. Cell Culture

HCT116 colon carcinoma and STF293 cells (human embryonal kidney-293 (HEK-293) cells stably transfected with the SuperTop-Flash Wnt-reporter containing seven LEF/TCF consensus binding sites driving firefly luciferase expression) [41] were grown in DMEM supplemented with 10% (*v*/*v*) fetal calf serum (PAN-Biotech, Aidenbach, Germany) and 1% (*v*/*v*) Pen/Strep solution (PAN-Biotech, Aidenbach, Germany) at 37 °C and 5% CO_2_. STF293 cells were kept under selective conditions with 0.1 mg/mL G418 (PAN-Biotech, Aidenbach, Germany). Wnt3a-conditioned medium and control medium were produced by Wnt3a-transfected and empty vector-transfected L-M-(TK-) cells, respectively, as described in [55].

### 4.3. Viability and Cytotoxicity Assays

HCT116 and STF293 cells were seeded in 96-well plates (1 × 10^4^/well). After 24 h, fresh medium was added. Five hours later, cells were treated with the different compounds at different concentrations, as indicated. After 20 h, cells were washed twice with PBS and subsequently incubated with 200 µL fluorescein diacetate (FDA) (Sigma, Taufkirchen, Germany) at a concentration of 10 μg/mL in PBS for 30 min at 37 °C. Released fluorescein was quantified with a Mithras LB940 microplate reader (Berthold Technologies GmbH & Co. KG, Bad Wildbad, Germany). Cytotoxicity of compounds was analyzed with the Cytotoxicity Detection Kit^PLUS^ (Roche, Mannheim, Germany), according to the manufacturer’s instruction, by measuring the release of lactate-dehydrogenase in response to damage of membrane integrity.

### 4.4. Cell Proliferation and Anchorage-Independent Growth Assays

Real-time monitoring of cell proliferation was performed using the xCELLigence system (Roche, Mannheim, Germany) as described previously [36]. Briefly, 7.500 HCT116 or STF293 cells per well were seeded on E-plates and grown for 24 h before addition of compounds at a final concentration of 3 μM, 10 μM or 30 μM. Changes in impedance were measured every 5 min at a frequency of 10 kHz and presented as the cell index (CI) calculated by the xCELLigence system software package.

Anchorage-independent growth of HCT116 cells was analyzed by colony formation in soft agar as described previously [56]. Assays were performed on 6-well plates with 2.500 cells/well in 1 mL of 0.3% (*w*/*v*) agar in phenol red-free DMEM. After 24 h incubation at 37 °C and 5% CO_2_, cells were treated with the indicated concentrations of the compounds diluted in 2 mL phenol red-free DMEM. Cells were grown for 21 days in the presence of compounds until single colonies were formed. After this incubation, colonies were fixed with ice-cold methanol for 60 min at −20 °C and stained with a 0.005% (*w*/*v*) crystal violet solution in 25% (*v*/*v*) methanol at room temperature. After destaining with repeated changes in Bidest. water, colonies were quantified with the Clono-Counter software [57]. Data were normalized to the DMSO control.

### 4.5. Reporter Gene Assays

STF293 cells were seeded into 96-well plates at a density of 1 × 10^4^ cells/well and left for 17 h for attachment before cells were pre-treated with Wnt3a/control-conditioned medium for 5 h. After 24 h treatment with the different compounds in the indicated concentrations, with DMSO as a vehicle control, cells were lysed in PPBT-buffer (100 mM K_3_PO_4_ pH 7.8; 0.2% (*v*/*v*) Triton X-100), and luciferase activities were measured using a Mithras LB940 microplate reader (Berthold Technologies GmbH & Co. KG, Bad Wildbad, Germany) as reported previously [58].

### 4.6. Western Blot Analyses

To analyze the expression levels of total β-catenin and active β-catenin, 8 × 10^5^ STF293 cells per well of a 12-well plate were seeded. After 24 h, medium was changed, and half of the medium volume was replaced by Wnt3a-conditioned or control-conditioned medium. Another 6 h later, cells were treated for 20 h with 3 μM HF, MC A, HM 177, HM 297 or DMSO (0.1% (*v*/*v*)) as a control. Subsequently, cells were lysed in lysis buffer (20 mM imidazole pH 6.8, 300 mM sucrose, 100 mM KCl, 2 mM MgCl_2_, 10 mm EGTA, 1 mM NaF, 1 mM Na_2_MbO_4_, 1 mM NaVO_3_, 0.5% (*v*/*v*) Triton X-100). After centrifugation (10 min, 20.800× *g*, 4 °C), to remove insoluble material, cell lysates were applied to SDS-PAGE and subsequently transferred to PVDF-membranes for detection of total β-catenin with anti-β-catenin (clone14; 1:1.000) antibody (BD BioSciences, Heidelberg, Germany) and active β-catenin with anti-active-β-catenin (anti-ABC clone 8E7; 1:1.000) antibody (Merck Millipore, Darmstadt, Germany). Anti-cyclinD1 (EPR2241, 1:2.000) antibody was obtained from Abcam (Cambridge, UK), anti-c-myc (N-262, sc-764X, 1:1.000) from Santa Cruz Biotechnology (Heidelberg, Germany), and anti-GAPDH (1:4.000) antibody from Merck Millipore (Darmstadt, Germany). HRP-labeled goat anti-mouse secondary antibodies were purchased from Dianova (Hamburg, Germany) and used in a dilution of 1:10.000 in TST-buffer (10 mM Tris/HCl pH 7.5, 150 mM NaCl, 0.1% (*v*/*v*) Tween 20). Active β-catenin signals were normalized to the GAPDH signal as a loading control. Chemiluminescence signals were detected using a G-Box imager (Syngene; obtained from VWR International GmbH, Darmstadt, Germany) and quantified with AIDA software (Rayest, Straubenhardt, Germany).

### 4.7. Quantitative RT-PCR

For analysis of the expression of endogenous Wnt target genes *Axin2* and *SP5*, 1 × 10^6^ HCT116 cells/well were seeded in 6-well plates. Medium was changed 24 h after seeding, and after 5 h, compounds were added at a concentration of 3 μM for 20 h. RNA was isolated using Nucleospin RNA II isolation kit (Machery & Nagel, Düren, Germany). Reverse transcription was performed using 1 μg total RNA applying the High-Capacity cDNA Reverse Transcription Kit (Life Technologies, Darmstadt, Germany). Quantitative PCR was performed on StepOnePlus Real-Time PCR System (Applied Biosystems, Darmstadt, Germany) using UPL probes and primers: *Axin2* UPL#60 (cat. No. 04688589001; Roche Life Sciences, Mannheim, Germany) with left primer 5′-CAAGCCTGGCTCCAGAAG-3′ and right primer 5′-GCATCCTCCGGRARGGAAT-3′; *SP5* UPL#36 (cat. No. 04687949001) with left primer 5′-GGGGAGACTCAGCAGACG-3′ and right primer 5′-TGGGTCCCTATGTCCGAAG-3′; *GAPDH* UPL#60 (cat. No. 04688589001) with left primer 5′-AGCCACATCGCTCAGACAC-3′ and right primer 5′-GCCCAATACGACCAAATCC-3′. Signals for Wnt target genes were normalized to *GAPDH*. Assays were performed in duplicate from 3 independent RNA isolations.

### 4.8. Statistical Analyses

Statistical analyses of all experiments and the creation of diagrams were performed with SigmaPlot 14.5 software (Systat, Erkrath, Germany). Data are presented as means ± SEM. Initially, normal distribution of values was tested by the method of Shapiro–Wilk. Normally distributed values were compared to another group by the two-tailed Student’s *t*-test (unless explicitly indicated otherwise in text/legends). In the case that a data set contained at least one non-normally distributed group, all comparisons were performed by the Mann–Whitney Rank sum test, which is indicated in the accompanying figure legends. For all tests, the significance criterion *p* < 0.05 was used. All numbers of replications (*n*) in figure legends refer to biological replicates.

## Figures and Tables

**Figure A1:**
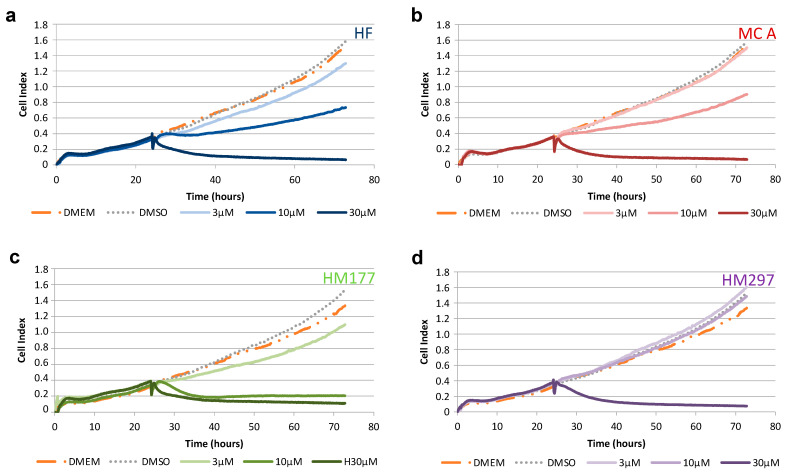
Analysis of cell proliferation of STF293 cells using real-time impedimetric measurement using the xCELLigence system in response to treatment with the indicated concentrations of (**a**) HF, (**b**) MC A and its derivatives (**c**) HM 177 and (**d**) HM 297. The graphs show representative cell index values of experiments performed in duplicate.

**Figure A2:**
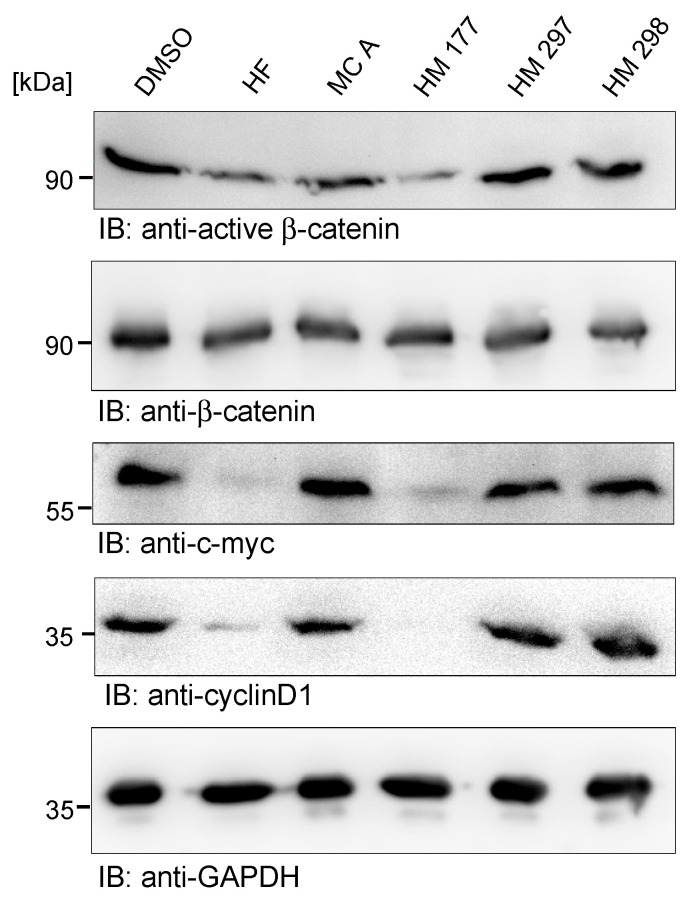
Active β-catenin is down-regulated in response to treatment with HF and MCs in HCT116 cells. Cell lysates were analyzed by SDS-PAGE and Western blotting, with the indicated antibodies. Interestingly, protein levels of the Wnt target genes *c-myc* and *cyclin D1* revealed a similar behavior. The image shows a representative Western blot of two independent blots.

## Data Availability

The data that support the findings of this study are available from the corresponding author upon reasonable request.
